# Knowledge and Perceptions of Diabetes in an Appalachian Population

**Published:** 2005-03-15

**Authors:** Irene Tessaro, Shannon L Smith, Sheila Rye

**Affiliations:** Department of Health Promotion and Risk Reduction, Community Health Initiatives; Department of Public Health Sciences, Wake Forest University School of Medicine, Winston-Salem, NC; Department of Health Promotion and Risk Reduction, School of Nursing, West Virginia University, Morgantown, WVa

## Abstract

**Introduction:**

Qualitative research on knowledge and perceptions of diabetes is limited in the Appalachian region, where social, economic, and behavioral risk factors put many individuals at high risk for diabetes. The aim of this study was to gain a culturally informed understanding of diabetes in the Appalachian region by 1) determining cultural knowledge, beliefs, and attitudes of diabetes among those who live in the region; 2) identifying concerns and barriers to care for those with diabetes; and 3) determining the barriers and facilitators to developing interventions for the prevention and early detection of diabetes in Appalachia.

**Methods:**

Thirteen focus groups were conducted in 16 counties in West Virginia in 1999. Seven of the groups were composed of persons with diabetes (n = 61), and six were composed of community members without diabetes (n = 40). Participants included 73 women and 28 men (n = 101).

**Results:**

Findings show that among this population there is lack of knowledge about diabetes before and after diagnosis and little perception that a risk of diabetes exists (unless there is a family history of diabetes). Social interactions are negatively affected by having diabetes, and cultural and economic barriers to early detection and care create obstacles to the early detection of diabetes and education of those diagnosed.

**Conclusion:**

Public health education and community-level interventions for primary prevention of diabetes in addition to behavior change to improve the management of diabetes are needed to reduce the health disparities related to diabetes in West Virginia.

## Introduction

West Virginia is a state with a disproportionately high burden of diabetes. During the last decade, West Virginia has consistently ranked among the highest diabetes prevalence and diabetes-related mortality of all the United States. In 1999, West Virginia had the third highest rate of death due to diabetes among all U.S. states (1) and ranked second in prevalence of diabetes with 7.6% of West Virginians having diabetes (2). Obesity, the most preventable cause of type 2 diabetes, has also been consistently higher in West Virginia than nationally; West Virginia has the second highest obesity rate in the nation (3).

The Appalachian region has a population with many social and health disparities contributing to the high rates of diabetes and obesity. High rates of poverty, low education, high unemployment, an aging population, and limited access to health care characterize many of the regions of Appalachia in general and West Virginia in particular (4). West Virginia is the second most rural state in the nation (5). Disparities in health status and risk factors among rural residents are well documented (6). Among all states, West Virginia has the nation's oldest population; nearly one third of West Virginians are older than 50 years (7). Additionally, 50 of West Virginia's 55 counties are designated as medically underserved (8). Of all states, West Virginia has the fourth highest percentage of adults aged 18 to 64 with no health care coverage (1).

Studies show that diabetes can be prevented or delayed in persons at high risk (primary prevention). For those with a high risk for diabetes, the benefits of interventions for physical activity and weight loss are clear (9,10). In the Diabetes Prevention Program, a large randomized clinical trial of 3234 individuals at 27 centers, a lifestyle intervention (e.g., physical activity, weight loss) was more effective than medicine in preventing or delaying the onset of diabetes in persons at high risk (10).

Community-wide public health interventions for physical activity and weight loss, community resources, and environmental changes can help reduce the morbidity and mortality from diabetes in West Virginia. Social and cultural factors are important elements in planning health promotion programs (11). Studies regarding the social and cultural influences on diabetes awareness and prevention that could help inform the design of community interventions are limited in the Appalachian region.

This paper presents data from focus group discussions with an underserved population of largely white, rural, aging participants in West Virginia, both those with and without diabetes. The existing qualitative research on lay perceptions and knowledge of diabetes focus on those with diabetes. Thus, studies of these perceptions before the onset of diabetes are also important to better understand cultural norms and beliefs about diabetes.

This research was a project of the Appalachian Diabetes Coalition based in West Virginia and funded by the Centers for Disease Control and Prevention. Its aim was to gain a cultural understanding of diabetes in the Appalachian region by 1) determining cultural knowledge, beliefs, and attitudes about diabetes; 2) identifying concerns and barriers to care for those with diabetes; and 3) determining the barriers and facilitators to developing interventions for the prevention and early detection of diabetes in Appalachia.

## Methods

### Data collection and sample

Thirteen focus groups were conducted in West Virginia over a five-month period in 1999. Seven groups were composed of 61 persons with diabetes, and six were composed of 40 community members without diabetes. All data were self-reported.

A sample of participants from 16 counties in six regions of the state was employed to represent all geographic areas of West Virginia. Research participant recruitment involved three steps: 1) designating communities for data collection, 2) identifying and involving community leaders, and 3) publicizing the focus group project on a local level to generate interest. Church leaders, activity and education coordinators, clinic and hospital staff, extension agents, and diabetes educators in the selected areas assisted with the recruitment process. Each focus group was conducted in a central community location as determined by community leaders who helped to organize the groups in their areas. Locations included health centers, clinics, hospital meeting rooms, churches, and senior centers (12).

Participants included 73 women and 28 men (n = 101) with a mean age of 59.1 years and 72.3% aged 50 or older. Participants with diabetes were on average older (64.5 years) than those without diabetes (53.3 years). The mean education level of all participants was 12.2 years, ranging from third grade to a completed master's degree. Twenty-seven participants (26.7%) did not complete a high school education. The mean education level of the participants with diabetes was 11.7 years, slightly less than the average of 12.9 years for those without diabetes. Overall, the sample was high-school educated. Most of the participants (94.1%) identified themselves as white, which reflects the population of West Virginia as a whole. According to 2000 census data, the population of West Virginia is predominantly (95%) white (13).

The authors moderated all focus groups. Groups were tape recorded, and a research assistant was present to take notes. Incentives to participants included health education print materials, a certified diabetes educator or health professional from the community to answer questions after the focus groups, and $25 to cover their time and transportation costs. The Institutional Review Board at West Virginia University approved the study.

Discussion questions were based on the explanatory model of illness (14), social learning theory (15), the health belief model (16), and social support theory (17). The discussion guide format elicited perspectives on diabetes and its management, including cultural knowledge, attitudes and beliefs about diabetes, prevention issues, early detection and health-seeking behavior, diabetes care issues, community health concerns, and information-seeking (Appendix). For those who did not have diabetes, the discussion guide was adjusted slightly to elicit general information helpful in interpreting cultural norms and attitudes about the disease. A brief eight-question survey was distributed at the beginning of each focus group to profile the participants demographically and assess the perception of risk of developing diabetes among participants from the general population.

### Data analysis

Focus group discussions were transcribed and verified with the handwritten notes. Transcripts were reviewed and imported into NUD*IST, a computer software package for qualitative data analysis (QSR International, Melbourne, Australia) (18). Qualitative methods were used to analyze data (19). Analysis began by coding the responses of the participants according to their contexts and relevance to the research question. Patterns arose during the systematic coding process, and themes were then determined by the researchers according to concepts and issues the participants emphasized repeatedly within groups and between groups. These themes are presented and illustrated with quotations from focus group participants. All quotations are taken from participants with diabetes unless [G] is indicated, in which case the quotation was provided by a general population participant without diabetes.

## Results

### Cultural beliefs and perceived susceptibility

Heredity, obesity, and physical inactivity were all recognized as risk factors for diabetes, with heredity often being mentioned in combination with another cause. Those with and without diabetes said that the disease most likely developed from inactivity (laziness) and lack of self-discipline (eating too much sugar). Because of these beliefs, blame and guilt were often associated with the diabetes diagnosis, along with a perception that diabetes was self-induced. Those with diabetes often recounted a specific period or event in their lives that they attributed to the development of the disease. Accounts of periods of inactivity and weight gain, pregnancy, stress, or a time where specific sugary foods were eaten were recalled. Respondents with the disease thought they could accurately identify their individual causes; however, without the classic risk factors of diabetes — overweight, a sedentary lifestyle, and particularly heredity — many discussants could not understand how they developed diabetes or why others with these risk factors did not have diabetes: "As far as I know, I'm the only one that had diabetes. None of my real blood kin has it. So why did I get it?"

A common belief about heredity was that diabetes only strikes every other generation of a family. Some participants felt that this was true for them or had heard about this happening to others: "Ours is hereditary, but it skips a generation. Not everybody gets it."

Through a short survey, participants in the general population (i.e., those without diabetes) focus groups were asked about their perceptions of risk of acquiring diabetes. As the Table shows, more than one third (35.9%) of participants did not know their risk. Almost half of those who had a family history of diabetes felt they were at high risk for developing diabetes, and no respondents without a family history of diabetes felt they were at high risk. Half of those without a family history of diabetes did not know their risk; the other half considered they were at small or average risk.

### Barriers to early detection

The inability to recognize symptoms of diabetes was cited as a major barrier to early detection and diagnosis. Participants generally perceived that if there were no recognizable symptoms, there was no need to go to a doctor or to think they were at risk. Not going to doctors was often mentioned as a barrier to early detection: "There is a lot of people that just never go to the doctor. . . . They were raised that way."

Participants felt that a lot of people did not want to know they had diabetes, particularly because it put a burden on the family. Transportation and inability to afford care were issues mentioned as general barriers to seeking care. For West Virginians, this is especially problematic because this state is the second most rural in the nation, with one of the lowest per capita incomes of all states: "I would say in a lot of rural areas, you have a lot of people who have an inability to go from point A to point B, just actually not being able to get there, period." [G]

Diabetes was considered a burdensome disease by most, a condition to be feared with severe complications. Some felt this fear was why people did not go to the doctor; people did not want to burden their family with their diabetes. There was also fear of the consequences of diabetes, especially amputation and blindness, as in the following example: "I think it's worse than cancer. I put it higher than cancer. Because it is long term. It's a slow process of dying. Where cancer seems to be more quick. . .where cancer, I hate to say it, it's not short and sweet. It's just short." [G]

### Knowledge about diabetes

Participants from the general population knew very little about diabetes, and those with diabetes knew little before diagnosis. Unless there was a family member with diabetes, there was little reason to be concerned or to have to know about diabetes. Most participants with diabetes never recognized symptoms before diagnosis and were diagnosed when under care for another health problem or on a routine visit.

Once diagnosed, participants reported they received little information from professionals to help them deal with the disease. They lacked knowledge in many areas — diet, physical activity, and resource information. Participants created alternative approaches to self-management according to their acquired knowledge about diabetes, which frequently appeared to be incorrect or incomplete. Participants felt they had caused the disease themselves, so the responsibility for controlling the disease fell heavily on them.

Participants consistently mentioned as issues lack of education by physicians about diabetes and lack of time spent with clients by physicians. They felt doctors knew little about nutrition, tended to prescribe medicine almost exclusively, and assumed people had money to pay for equipment, such as test strips. Additionally, they felt like they were being rushed and often forgot what they wanted to ask about their diabetes. Doctors did not usually explain what prescribed medicine was for or what the side effects could be.

The cost of care was another major concern for individuals with diabetes. Many felt physicians did not understand or were unwilling to deal with cost issues:

"There is lots of things I have go wrong that I need to tell the doctor. But I know that I can't go out here and pay for all these tests, so I will keep it to myself. I don't even tell him because I know he's going to want extensive blood work of this or that and I don't do it, so I keep it to myself."

"I told my doctor the same thing. If you do not give me my medicine, there is no way I can afford the medicine. I will just have to die early I reckon."

Participants with diabetes wanted more information about their condition but often did not know where to go to get it. Dietitians and diabetes educators offered information when they were available. However, few diabetes educators or nutritionists are located in West Virginia, particularly in rural areas. Participants felt few affordable health professionals or educational programs could give them the information they needed to deal with their diabetes. Some participants sought information from members of their social networks, the pharmacy, or the health department. Many relied on magazines for new information. Almost all had access to a general practitioner, but few to diabetes specialists.

Although persons with diabetes seemed to recognize the relationship between physical activity and blood glucose control, very little discussion of actual exercise behaviors generated around it. Lack of energy from diabetes, the cost associated with exercise because of the need to check blood levels before and after exercise, and lack of community resources were cited as barriers to exercise: "There again is a vicious circle because the more you sit around, the fatter you get. You eat and you sit some more and you just keep going in the circle." Although weight loss was readily identified as a means of controlling the disease, the focus remained on methods other than physical activity, such as a reduced-calorie or reduced-fat diet.

### Social relationships

Participants with diabetes discussed the effect the disease had on their social relationships. Because of certain beliefs related to diabetes causation, particularly laziness and eating too much of the wrong foods, diabetes was perceived as a self-induced disease. The stigma associated with a disease perceived as self-induced and not under control, along with the perception that others feel the same way, led to participants' concern that others treated them differently. They also expressed feeling depressed because others did not understand how diabetes was affecting them. Having support from others with diabetes who are dealing with similar issues was mentioned by participants as something that could help them cope with diabetes.

Furthermore, a number of participants expressed that others may not really think they are sick because they do not look sick:

"You know, I think part of the reason people think that way is because we do look healthy. I mean, we don't look any different. You can't see any problem that we have. Diabetes is usually a slow acting disease. You know it takes years and years before you see anything bad come from it."

The effect of diabetes on social relationships was not limited to those with diabetes. Participants from the general population focus groups felt that at times they did treat persons with diabetes differently:

"You find yourself getting short tempered. You get irritated real easy sometimes. You get irritated with other people because they have to eat on time; they take their shots. You get into a restaurant and here they've taken their meds waiting on their food because it has to be in their blood system for at least 30 minutes. They're just taking their time. Yeah, you get irritated [with a person with diabetes]." [G]

## Discussion

This qualitative research was conducted to gain insight into the cultural understandings surrounding diabetes in West Virginia. Participants lacked knowledge about diabetes before and after diagnosis and had little perception of risk of diabetes other than a family history of diabetes. Social interactions were reported to be negatively affected by having diabetes, and cultural and economic barriers to early detection and care create obstacles to the early detection of diabetes and education once diagnosed.

Poverty is an integral component of West Virginian culture, and economic circumstances dictate many behaviors. In a resource-poor area such as West Virginia, individuals adopt creative strategies for coping with diabetes because they lack access or have only limited access to diabetes education, health care, health care providers with knowledge to educate patients about diabetes and its management, exercise facilities, and the types of food needed (20). Decisions about early detection of diabetes and care-seeking are frequently made from the integration of cultural values with the pervading poverty. Socioeconomic factors appear to be major influences on health-related decision making, creating disparities in the diagnosis and treatment of diabetes in this Appalachian sample.

Our focus groups found that the health care system provided little information to persons diagnosed with diabetes, making it difficult for those persons to find affordable education to manage diabetes. Participants reported that their physicians knew little about the disease. Those with diabetes lacked knowledge about diet, physical activity, and resource information. It was clear from the focus groups that persons with diabetes needed information tailored to their individual needs, whether it be medication, health behavior change, or coping with a diagnosis of diabetes.

Because of its chronic nature, diabetes requires daily management to control blood glucose levels, including a dietary regimen, regular exercise, routine monitoring of blood glucose levels, regular physician office visits, and for some, daily hypoglycemic agents. A person with diabetes adjusts and adapts to these modifications and restrictions within the framework of his or her cultural influences, economic circumstances, knowledge, and resources, regardless of clinical recommendations (21). Because the concerns of the patient and practitioner surrounding illness and treatment often differ, discord between clinical and lay models is often medically labeled as nonadherence (22,23). What is medically labeled as nonadherent behavior, however, is often a common-sense adaptation for the patient from within his or her belief framework, cultural context, and outside influences, such as financial constraints, limited knowledge, and lack of availability of appropriate medical care and/or facilities. Without knowledge about their disease, nutritional education, and access to affordable and/or appropriate items, people often rely on information that is partial, outdated, or incorrect (23). If persons are not satisfied with the information they are receiving from their health care providers, they will seek alternative sources to help them manage their disease. In short, lack of knowledge and high costs hamper preventive health behaviors in rural Appalachia (24).

The stigma associated with diabetes because of self-blame has been shown to negatively affect social relationships. Cohen et al (21) found that managing diabetes was a major obstacle to social relationships, contributing to such events as divorce, loss of jobs, sexual problems, and infertility. Maclean (22) found that those with diabetes were very concerned about how others would treat them once they discovered they had diabetes, so they avoided situations in which they were likely to be treated differently. The current study found that while those with diabetes did not want to be treated differently, they also felt others did not understand what they were going through. Stigma is a social label, defined within a cultural context, that changes the way individuals view themselves and are viewed by others (25). Persons with diabetes are "blamed" for their disease because of the perception of responsibility both on the part of those with and without diabetes. It is not surprising, then, that support from others with diabetes who were dealing with similar issues was repeatedly brought up as an important strategy for change in these focus groups.

### Implications for public health

Several findings of this study point to the need for more public health education and community-level interventions for primary prevention of diabetes and behavior change to improve the management of diabetes and to reduce the health disparities related to diabetes in West Virginia. Primary prevention of diabetes is becoming more important because of findings from the lifestyle modification prevention trials (26). Health promotion programs that combine both behavioral and social and physical environmental change strategies can provide a more comprehensive approach to addressing diabetes (27). Several behavioral and environmental intervention strategies have the potential for the primary prevention of diabetes in addition to behavioral change for those with diabetes in rural West Virginia. Health communications that address a person's unique situation and personal characteristics have been shown effective for changing health behaviors for a variety of health issues and populations (28). Social network interventions, such as lay or natural helpers, build on the naturally existing sources of social and community support to diffuse health information and provide support for behavioral and social change (29). For those with diabetes, support and health information, through formal or informal groups, can be beneficial (30). Group medical visits have been suggested for some chronic diseases and could be an effective strategy to provide support as well as education and disease management (31).

## Figures and Tables

**Table:** Perceived Risk for Diabetes Among Study Participants Without the Disease, West Virginia, 1999^a^

**Perceived Risk**	**Family History of Diabetes (n=21)**	**No Family History of Diabetes (n=18)**	**Total (N=39)**
High	10 (47.6)	0 (0)	10 (25.6)
Average	5 (23.8)	4 (22.2)	9 (23.1)
Small	1 (4.8)	3 (16.7)	4 (10.3)
No risk	0 (0)	2 (11.1)	2 (5.1)
Don’t know risk	5 (23.8)	9 (50.0)	14 (35.9)

a Values are numbers (percentages). N = 39 because responses on family history of diabetes and perceived risk were not provided by one of the 40 participants.

## Appendix: Diabetes Focus Group Scripts

### Persons Without Diabetes (focus group script)

**Cultural knowledge, beliefs, and attitudes about diabetes**

- What comes to mind when you think about diabetes?
- How much of a concern is diabetes for you personally?
- What concerns you most about diabetes?*

- What do you know about diabetes?
- What do you think causes diabetes?*
- Who are the people most at risk for developing diabetes?*
- When does diabetes develop?*
- What are the symptoms or signs of diabetes?
- What are some of the problems diabetes can cause?

- Do you know persons who have diabetes?
  - How has diabetes affected their life?*
    - Probe: problems, reactions of others

**Prevention and early detection of diabetes**

- What prevents people from finding out they have diabetes?*
- What do you think is the best way to detect diabetes early?*

- What do you think people can do to prevent getting diabetes?*
- What behavior changes can one make to prevent diabetes?*
  - What information or help would you need to make these behavior changes?

- Do you think people need more information about diabetes?*
  - What kind of information would be most needed?*
- What is the best way to get health information to people about diabetes and early detection?*
  - Probe: community, church, medical, media, print materials

### Persons With Diabetes (focus group script)

**Cultural knowledge, beliefs, and attitudes about diabetes**

- What concerns you most about having diabetes?*
- How has diabetes affected your life?*
  - Probe: problems, reactions of others, behavior change

- What do you think causes diabetes?*
- Who are the people most at risk for developing diabetes? *
- When does diabetes develop?*

**Prevention and early detection of diabetes**

- What prevents people from finding out they have diabetes?*
- What do you think is the best way to detect diabetes early?*

- What do you think people can do to prevent getting diabetes?*
- What behavior changes can one make to prevent diabetes?*

- Do you think people need more information about diabetes?*
  - What kind of information would be most needed?*
- What is the best way to get health information to people about diabetes and early detection?*
  - Probe: community, church, medical, media, print materials

**Diabetes care issues**

- What are the ways you take care of your diabetes?
  - Probe: treatment (biomedical), behavior change, alternative/folk medicine
  - Probe: advantages, disadvantages

- What concerns do you have about getting health care for your diabetes?
  - What problems have you encountered getting care and treatment?

- What do you want to know about diabetes that you don’t already know?
- What kind of information would help you most with your diabetes?
- What is the best way to get this information to you?
  - Probe: community, church, medical, media, print materials

* same question in both groups
